# Functional availability of medical oxygen for the management of hypoxaemia in Cameroon: A nationwide facility-based cross-sectional survey

**DOI:** 10.7189/jogh.14.04092

**Published:** 2024-05-10

**Authors:** Yauba Saidu, Agbor Valirie Ndip, Ousmane Diaby, Bonaventure Hollong, Zachary Katz, Audrey Battu, Sangwe Clovis Nchinjoh, Adamou Dodo Balkissou, Owens Wiwa

**Affiliations:** 1 Clinton Health Access Initiative Inc., Yaounde, Cameroon; 2 Institute for Global Health, University of Siena, Siena, Italy; 3 Clinical Trial Service Unit and Epidemiological Studies Unit (CTSU), Nuffield Department of Population Health, University of Oxford, Oxford, England, UK; 4 Ministry of Public Health, Yaounde, Cameroon; 5 Global Essential Medicines, Clinton Health Access Initiative Inc., Boston, Massachusetts, USA; 6 Hôpital Régional De Garoua, BP 121 Garoua, Cameroon

## Abstract

**Background:**

Medical oxygen is essential for managing hypoxaemia, which has a multifactorial origin, including acute and chronic lung diseases such as pneumonia, asthma, and severe malaria. The coronavirus disease 2019 (COVID-19) revealed substantial gaps in the availability and accessibility of safe medical oxygen, especially in low- and middle-income countries (LMICs). This study aimed to assess the availability and sources, as well as the barriers to the availability of functional medical oxygen in hospitals in Cameroon.

**Methods:**

This was a nationwide cross-sectional descriptive study conducted from 26 March to 1 June 2021. Using a convenient sampling technique, we sampled accredited public and private COVID-19 treatment centres in all ten regions in Cameroon. Representatives from the selected hospitals were provided with a pre-designed questionnaire assessing the availability, type, and state of medical oxygen in their facilities. All analyses were performed using R.

**Results:**

In total, 114 hospitals were included in this study, with functional medical oxygen available in 65% (74/114) of the hospitals. About 85% (23/27) of the reference hospitals and only 59% (51/87) of the district hospitals had available functional medical oxygen. Compared to district hospitals, reference hospitals were more likely to have central oxygen units (reference vs. district: 10 vs. 0%), oxygen cylinders (74 vs. 42%), and oxygen concentrators (79 vs. 51%). The most common barriers to the availability of medical oxygen were inadequate oxygen supply to meet needs (district vs. reference hospitals: 55 vs. 30%), long delays in oxygen bottle refills (51 vs. 49%), and long distances from oxygen suppliers (57 vs. 49%).

**Conclusions:**

The availability of medical oxygen in hospitals in Cameroon is suboptimal and more limited in districts compared to reference hospitals. The cost of medical oxygen, delays related to refills and supplies, and long distances from medical sources were the most common barriers to availability in Cameroon.

Medical oxygen is essential for managing hypoxaemia, which indicates abnormally low levels of oxygen in the blood. Hypoxemia is a life-threatening condition that can be a complication or feature of diseases such as pneumonia, heart failure, and asthma. Medical oxygen is also important in managing patients undergoing surgery, critically ill patients, and preterm babies to prevent hypoxemia. In 2017, the World Health Organization (WHO) included medical oxygen on the Model List of Essential Medicines and the List of Essential Medicines for Children to manage hypoxemic medical conditions such as pneumonia, asthma, and heart failure to avoid preventable disabilities and deaths [1,2].

Globally, severe pneumonia is the leading infectious cause of death among children. In 2015, severe pneumonia was responsible for 22 million hospitalisations among children younger than five [3]. In low- and middle-income countries (LMICs), severe pneumonia leaves about 4.2 million children less than five years old with severe hypoxaemia every year [4]. In 2017, severe pneumonia was responsible for about 800 000 deaths among children under five years old from LMICs, 20–40% of which could have been prevented with the availability of medical oxygen [5]. Additionally, pneumonia-related deaths in young children are higher than those caused by malaria, diarrhoea, and measles combined.

Despite the burden of pneumonia and the need for medical oxygen in LMICs, approximately half of the health facilities in resource-limited settings had either no or inconsistent supply of medical oxygen and pulse oximeters [6–8]. The situation is worst in the poorest countries, especially those in sub-Saharan Africa (SSA), where under 20% of health facilities have the medical oxygen they need. In Nigeria, only about 10% of children with severe pneumonia received the medical oxygen they required [8]. In addition, most hospitals in rural areas in Kenya lack the supplementary materials and a reliable electricity supply needed to supply medical oxygen [9]. Furthermore, these hospitals struggle with adequate supply chains for medical oxygen cylinders [9]. Even when available, the cost of medical oxygen is unaffordable for most patients from low-income households in SSA, which results in health inequalities in the most vulnerable and poorest populations. It is estimated that the cost of a three to four day treatment of a child with severe pneumonia varies between 40–60 US dollars (USD) [10] and could be as high as 100 USD in Somalia [11].

The coronavirus disease 2019 (COVID-19) exacerbated the global demand for medical oxygen, highlighting the extent of the challenges related to the inaccessibility and unaffordability of medical oxygen in SSA. Pneumonia is a key feature of severe COVID-19, and supportive therapy with medical oxygen is crucial for the survival of patients. The WHO estimated that about 5% of patients with COVID-19 will be critical and will require oxygen supplementation in an intensive care setting [12]. In LMICs alone, the need for medical oxygen has increased to about 1.1 million cylinders [5].

Within the context of Partnership for Maternal, Newborn & Child Health ‘Call to Action on COVID-19’, the WHO calls for health systems to invest in medical oxygen to ensure safe, equitable, and efficient production, distribution, and management of oxygen [13]. In order to understand the need for medical oxygen in Cameroon, this study sought to assess the functional availability of medical oxygen in district and regional hospitals in Cameroon.

## METHODS

### Study design and participants

This nationwide cross-sectional descriptive study was conducted from 26 March to 1 June 2021 by a Technical Working Group instituted by Cameroon’s Ministry of Health. Cameroon is a Central African country with about 27 076 679 inhabitants, 50.4% of whom are females [14]. Cameroon consists of 10 administrative regions. The health system pyramid in Cameroon is organised into three levels. The peripheral (operational) level that contains district hospitals forms the base of the pyramid and is involved with care provision, district coordination, and regulation. The intermediate level provides technical support to the peripheral level and is responsible for regional coordination, regulation, and supervision. It consists primarily of regional hospitals and hospitals with similar technical platforms as well as specialised regional hospitals. The central level, which contains general and central hospitals, forms the top of the pyramid and is responsible for developing, regulating, and coordinating health strategies. In 2020, there were seven COVID-19 treatment centres, six general hospitals, nine central hospitals, 15 regional hospitals, and 290 district hospitals in Cameroon.

### Study participants

All accredited public and private district and reference hospitals within the ten regions of Cameroon were considered for inclusion. For this study, reference hospitals consisted of all facilities at the intermediate and central levels of Cameroon’s health system, including general, central, and regional hospitals and specialised COVID-19 treatment centres. We invited representatives of all districts and regional hospitals in Cameroon to provide information on the availability of medical oxygen and related accessories like cylinders and oxygen concentrators in their respective facilities. The director of each health facility was contacted through phone calls to send a representative from their facility to the regional delegation for health to participate in the survey. Hospitals that did not send a representative were excluded from the study.

### Sampling and sample size

We targeted 130 reference and district hospitals from all ten regions in Cameroon. We recruited facilities using a convenient sampling technique.

### Study procedure and data collection

Authorisation to conduct this study was granted by Cameroon’s Ministry of Health. Trained data collectors from each regional delegation administered the data collection form to participants as a structured Microsoft Office Excel Form. The data collectors were available to clarify participants’ doubts or misunderstandings related to the questionnaire.

We collected information on the role of the respondent within the health facility, type of health facility, region of location of the facility, prior evaluation of the state of medical oxygen in the health facility, and availability of oxygen within the facility.

In order to assess the functional availability of medical oxygen, respondents were asked if their facility provided medical oxygen to patients in need, such as those with hypoxemia. Information was also collected on the source of oxygen distribution (central oxygen unit, oxygen cylinder, or oxygen concentrators) and the availability of accessories for medical oxygen, such as nasal cannulas, wall dispensers, and high- and low-flow oxygen masks. Finally, we asked respondents what they think might be barriers to the availability of medical oxygens, including the cost of oxygen bottles, availability of oxygen bottles, duration of oxygen bottle refills, adequacy of oxygen supply to meet needs, and long distance from supplier.

### Data management and analysis

We exported collected data to the statistical software R (version 3.5.1, 2019, The R Foundation for statistical computing, Vienna, Austria) for data cleaning, analysis, and visualisation. Frequencies and percentages were used to summarise categorical variables. Chloropleth maps and bar plots were used for data visualisation.

## RESULTS

### Characteristics of health facilities included in the study

**Figure 1:**
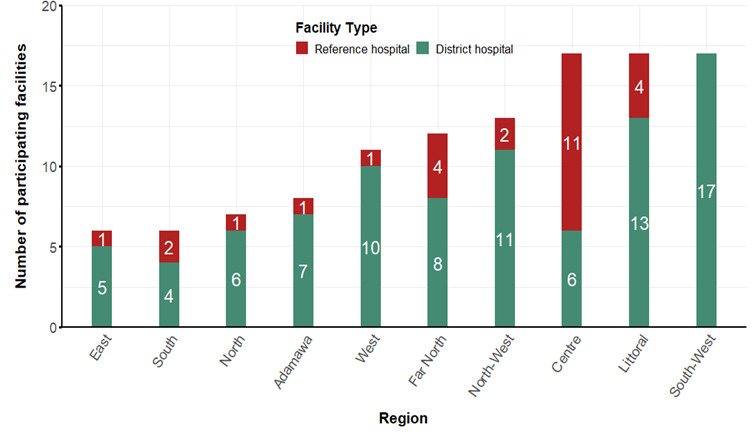
Number of participating health facilities by region.

A total of 114 hospitals were enrolled in the survey from all ten regions, 87 (76.3%) of which were district hospitals. In contrast, 27 (23.7%) were reference hospitals (12 regional hospitals, ten central hospitals, and five general hospitals. The majority of the participating hospitals were from the Centre, Littoral, and South-West regions (**Figure 1**).

The respondents of the questionnaire were mostly hospital administrators (74 (65.5%)), who included directors, medical advisors, general supervisors, and district medical officers (**Table 1**). Only about 5% and 22% of district hospitals and reference hospitals had undergone prior evaluations regarding the availability of medical oxygen within their facilities, respectively (**Table 1**).

**Table 1:** Characteristics of health facilities included in the study

Characteristics	Type of facility		Total
	District (n = 87)*	Reference (n = 27)*	
Role of respondent			
*Hospital administrator*	58 (67.4%)	16 (59.3%)	74 (65.5%)
*Medical doctor*	8 (9.3%)	2 (7.4%)	10 (8.8%)
*Anaesthetist/Nurse anaesthetist*	2 (2.3%)	4 (14.8%)	6 (5.3%)
*Others*	18 (20.9%)	5 (18.5%)	23 (20.4%)
Prior evaluation for medical O_2_ availability	4 (4.6%)	6 (22.2%)	10 (8.8%)

*N = frequency.

### Availability of medical oxygen in Cameroon

**Figure 2:**
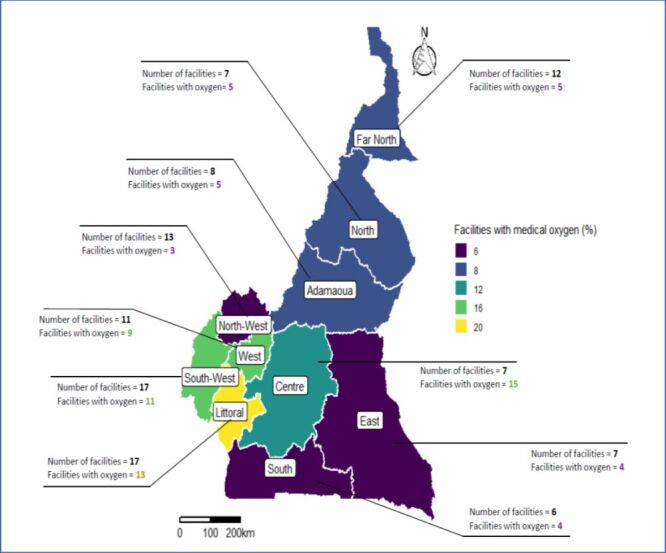
Availability of health facilities with medical oxygen across all ten regions in Cameroon.

In total, 74 (64.9%) of the 114 hospitals reported having functional medical oxygen. Functional medical oxygen was available in 23 (85.2%) of the 27 reference hospitals and only 51 (58.6%) of the 87 district hospitals. The Littoral, Centre, South-West, and West regions had the highest number of facilities with medical oxygen. In contrast, the East, South, and North-West regions had the lowest number of health facilities with medical oxygen (**Figure 2**).

### Availability of different sources for oxygen delivery

**Figure 3:**
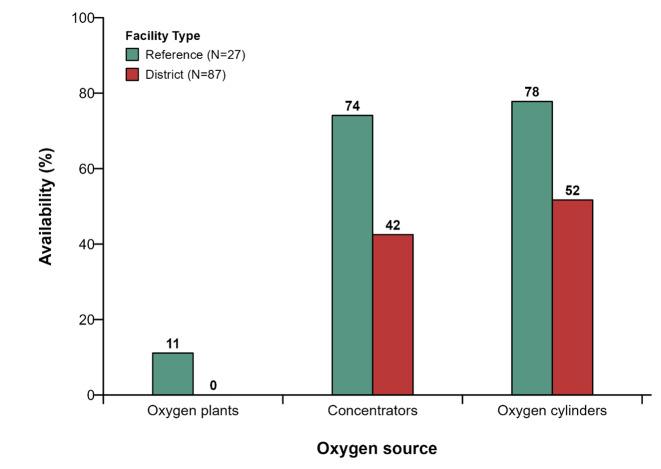
Availability of Sources of oxygen by type of facility in Cameroon.

The three major sources of oxygen distribution in this study were a central oxygen unit, oxygen cylinders, and oxygen concentrators (**Figure 3**). Central oxygen units were rarely available in both reference and district hospitals. No district hospital had a central oxygen source, while only three (11.1%) of the 27 reference hospitals had a central oxygen source.

Only about 42 and 51% of the district hospitals had oxygen cylinders or concentrators, respectively (**Figure 3**). Meanwhile, 74 and 76% of the reference hospitals had oxygen cylinders and concentrators, respectively (**Figure 3**).

### Availability of supplementary materials for medical oxygen administration

**Figure 4:**
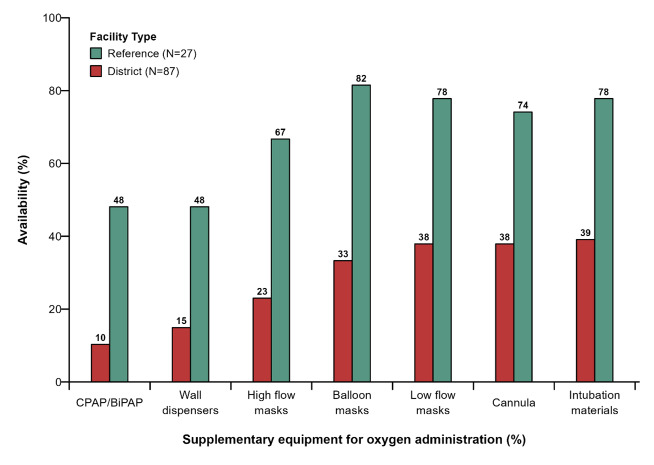
Availability of accessories for the administration of medical oxygen.

Less than 40% of district hospitals had supplementary materials for medical oxygen administration, Continuous Positive Airway Pressure (CPAP), Bilevel Positive Airway Pressure (BiPAP), wall dispensers, and high-flow oxygen masks were rarely available (**Figure 4**).

Over 70% of the reference hospitals had balloon masks, materials for endotracheal intubation, low-flow masks, and cannula. However, under 50% of reference hospitals had CPAP/BiPAP and wall dispensers.

### Barriers to the availability of and accessibility to medical oxygen

**Figure 5:**
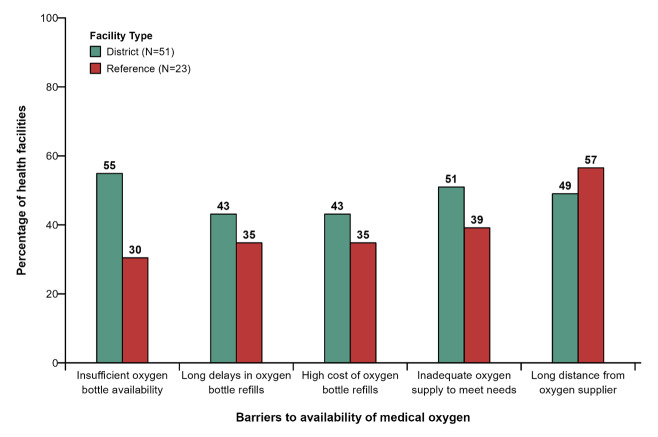
Barriers to the availability of medical oxygen. The numbers on the bars are the number of facilities where these accessories are available.

We assessed barriers to the availability of and accessibility to medical oxygen among the 23 reference and 51 district hospitals that declared having functional medical oxygen. Overall, district hospitals faced more difficulties obtaining medical oxygen than reference hospitals (**Figure 5**). Inadequate supply of medical oxygen in relation to demand, long delays in oxygen bottle refills, and long distance from the facility to the supplier were the major barriers to the availability of medical oxygen within facilities included in this survey (**Figure 5**).

## DISCUSSION

This study found that functional medical oxygen was available in about 65% of district and reference hospitals. The availability of medical oxygen and supplementary materials for medical oxygen delivery was significantly lower in district than in reference hospitals. There were regional inequalities in the distribution of medical oxygen, with the East, South, and West regions of Cameroon having the lowest number of hospitals with functional medical oxygen. Oxygen cylinders and oxygen concentrators were the primary sources of oxygen delivery in district and reference hospitals, while central oxygen units were scarce. Materials for intubation, low-flow oxygen mask, and cannula were the most common supplementary materials for administering oxygen. The availability of these materials was twice as common in reference than in district hospitals. Finally, inadequate supply of medical oxygen in relation to demand, long delays in oxygen bottle refills, and long distance from the facility to the supplier were the significant barriers to the availability of medical oxygen within facilities.

Our finding was similar to a study conducted on 32 health facilities in Ethiopia, which found that medical oxygen was available in just 62% of these facilities [15]. Medical oxygen is essential for the management of patients with hypoxaemia [1,2], a medical emergency that should be managed within minutes, depending on the severity. For example, permanent brain damage begins within four minutes in an unconscious patient who is not breathing, and death can ensue within eight minutes. Hypoxaemia is a feature of many medical conditions, such as pneumonia, congestive heart failure, severe malaria, chronic respiratory diseases, tuberculosis, and prematurity, which are significant contributors to disabilities and deaths in Cameroon [16]. Therefore, ensuring the availability of functional medical oxygen is vital in managing these patients to reduce preventable morbidity and mortality related to hypoxemia in Cameroon.

Several factors could explain the regional inequalities in the availability of medical oxygen in Cameroon. Medical oxygen from oxygen plants in Douala, the capital of the Littoral region, needs to be bottled in oxygen cylinders for distribution to other regions. Consequently, the distance from the health facility to the oxygen source poses a considerable challenge to the regional distribution of medical oxygen in Cameroon. Moreover, regions with mountainous topography or poor road networks, such as the North West Region, were less likely to receive functional medical oxygen [17]. Furthermore, a lack of or sub-optimal electricity supply, as well as a lack of staff with expertise in oxygen administration, could be responsible for regional inequalities in the availability of functional oxygen in Cameroon [18]. Moreover, the cost of medical is a major barrier to the availability of functional oxygen as most district hospitals cannot afford sufficient oxygen to meet care demands.

Due to the emergency associated with hypoxaemia management, it is even more crucial for functional medical oxygen to be available in all district hospitals, which are usually the first point of referral from sub-divisional hospitals. However, we found that functional medical oxygen was only available in about 69% of district hospitals. Moreover, supplementary materials for administering medical oxygen were less common in district than in reference hospitals. A study conducted in Kenya found low availability of supplementary materials for medical oxygen delivery, especially within facilities located in rural areas [9]. Improving access to and availability of medical oxygen in district hospitals is vital to reducing the morbidity and mortality related to hypoxaemia.

A study conducted in Ethiopia suggests that scarcity in functional medical oxygen is amendable using multifaceted approaches such as capacity-building on the maintenance of oxygen devices in health facilities, providing technical support and supportive supervision to the ministries of health and regional delegations for health, and provision of medical oxygen devices such as concentrators, oxygen cylinders, and wall dispensers [15]. In addition to improving the availability and distribution of medical oxygen devices, ensuring the effective supply of oxygen over the national territory and subsidising the cost of medical oxygen and supplementary materials could go a long way to improve the availability of medical oxygen in Cameroon.

### Study limitation

The use of a non-probabilistic sampling technique limits the generalisability of our findings to Cameroon. However, our sample included about 72 and 30% of Cameroon’s reference and district hospitals, respectively. Moreover, our sample covered hospitals in all ten regions in Cameroon. As a result, our findings reflect, to a greater extent, the reality of the state of functional medical oxygen in Cameroon. Nonetheless, this study might have overestimated the availability of functional medical oxygen because the presence of functional medical oxygen was reported by hospital representatives rather than objectively assessed through an audit.

## CONCLUSIONS

We found that the availability of functional medical oxygen and supplementary materials for its administration was suboptimal in district hospitals in Cameroon. Moreover, the cost of medical oxygen and obtaining medical oxygen supplies, bottle refills, and long distances between the facility and source of oxygen were the major barriers to medical oxygen in Cameroon.
